# Impact of implementation of a ventilator-associated pneumonia prevention bundle that includes selective digestive decontamination in a southern region of Spain

**DOI:** 10.1186/2197-425X-3-S1-A705

**Published:** 2015-10-01

**Authors:** S Rebollo, R Jiménez, A Ortín, E Andreu, B Gil, MA López, M Royo-Villanova, L Capilla, JJ Rodríguez, A Martínez-Pellús

**Affiliations:** Hospital General Universitario Santa Lucía, Servicio de Medicina Intensiva, Cartagena, Spain; Hospital Universitario Virgen de la Arrixaca, Servicio de Medicina Intensiva, Murcia, Spain; Hospital Universitario J.M. Morales Meseguer, Servicio de Medicina Intensiva, Murcia, Spain; Hospital General Universitario Reina Sofía, Servicio de Medicina Intensiva, Murcia, Spain

## Introduction

Ventilator-associated pneumonia is a common infection in Intensive Care environment, with a high attributable morbidity and mortality. In recent years, several strategies of prevention have demonstrated benefit.

## Objectives

To describe the impact of a VAP prevention bundle that includes selective digestive decontamination (SDD) in our setting.

## Methods

On June 2010, a VAP prevention bundle was implemented in province of Murcia (Spain) This bundle included: appropriate airway secretions management, strict hand hygiene, cuff pressure control, oral hygiene with chlorhexidine, semi-recumbent positioning and selective decontamination of the digestive tract (SDD) with a short course of parenteral antibiotic.

On april 2011 was implemented “Neumonía Zero” project at National level, which includes those same recommendatios but with SDD only as an optional highly recommended measure.

We collect data from four public health system hospitals, before (january 2006 to June 2010) and after (july 2010 to december 2014) the implementation of the bundle.

## Results

In the preimplantation period 9924 patients was analyzed, with 19924 days of mechanical ventilation (MV). In the period after the implementation of the bundle, 17060 patients and 40044 days of MV was studied.

VAP rate passed from a mean of 15.1 cases per 1000 ventilator-days, to 8.8, 6.4 and 5.4 cases per 1000 ventilator-days in the three consecutive 18-months periods after the introduction of the bundle.

We analyzed the etiology of VAP across time. P. aeruginosa was the causal bacteria in 30.5 % of cases in preimplementation period and 32.5 % in postimplementation one. S. Aureus passed from 11.5 to 9.3 %; A. baumanii from 3.6 to 4.2%; Other enterobacteriae from 38 to 26.1 %; and other etiologies from 18.8 to 27.7 %.

## Conclusions

Introduction of a ventilador-associated pneumonia prevention bundle that includes selective digestive decontamination led to a progressive reduction in our high rate of VAP. No clear evidence of change in microbiological etiology has been observed as a result of initiation of selective digestive decontamination.
